# Optimization of Primary Care Among Black Americans Using Patient Portals: Qualitative Study

**DOI:** 10.2196/27820

**Published:** 2021-06-03

**Authors:** Omar H Ordaz, Raina L Croff, LaTroy D Robinson, Steven A Shea, Nicole P Bowles

**Affiliations:** 1 Oregon Institute of Occupational Health Sciences Oregon Health Sciences University Portland, OR United States; 2 Department of Neurology School of Medicine Oregon Health Sciences University Portland, OR United States

**Keywords:** health promotion, patient engagement, telehealth, telemedicine, health disparities, technology acceptance model, health belief model

## Abstract

**Background:**

Reduced patient portal use has previously been reported among Black Americans when compared with that of the general population. This statistic is concerning because portals have been shown to improve the control of chronic conditions that are more prevalent and severe in Black Americans. At their very simplest, portals allow patients to access their electronic health records and often provide tools for patients to interact with their own health information, treatment team members, and insurance companies. However, research suggests that Black American patients have greater concerns over a lack of support, loss of privacy, and reduced personalization of care compared with other Americans, which results in a disparity of portal use.

**Objective:**

This qualitative investigation of primary care experiences of Black Americans from across the United States who participated in remote focus groups in April and May 2020 aims to explore the use and perceived value of patient portals to better understand any barriers to optimized treatment in the primary care setting.

**Methods:**

We performed an inductive thematic analysis of 8 remote focus group interviews with 29 Black American patients aged 30-60 years to qualitatively assess the experiences of Black American patients with regular access to portals.

**Results:**

Thematic analysis uncovered the following interrelated themes regarding patient portals in primary care: the optimization of care, patient empowerment, patient-provider communication, and patient burden.

**Conclusions:**

In contrast to what has been described regarding the reluctance of Black Americans to engage with patient portals, our focus groups revealed the general acceptance of patient portals, which were described overwhelmingly as tools with the potential for providing exceptional, personalized care that may even work to mitigate the unfair burden of disease for Black Americans in primary care settings. Thus, opportunities for better health care will clearly arise with increased communication, experience, and adoption of remote health care practices among Black Americans.

## Introduction

### Background

The use of electronic health records (EHRs) is rapidly becoming the norm for primary care services. The American Recovery and Reinvestment Act of 2009 attempted to provide access to EHRs for every American within 5 years through US $19 billion in incentives in the Health Information Technology for Economic and Clinical Health Act [1]. According to the most recent National Electronic Health Records Survey from the Centers for Disease Control and Prevention in 2017, 85.9% of office-based doctors use EHRs—up by 37.6% from the year the Health Information Technology for Economic and Clinical Health Act was passed [2,3].

Patient portals, which were first developed by software vendors and health care centers in 1999, provide patients access to their medical charts and other data from their EHRs [4]. Patient portals vary by provider but many also facilitate payments, scheduling, and medication refills; link patients to educational materials; and allow password-secured communications with providers [5,6]. These tools are associated with increased effectiveness in the comprehensive care of chronic diseases, including diabetes and hypertension [7], which are both more prevalent and result in more severe outcomes among Black Americans compared with the rest of the American population. This health disparity is likely due to the psychosocial and socioeconomic stresses associated with the structural racism that is evident across the United States [8]. Proactive, portal-based secure messaging from the treatment team to the patient between visits has been shown to increase patient engagement and self-management behaviors in the treatment of chronic disease [9]. Despite the rapid adoption of patient portals at clinics and the positive outcomes associated with patient portal use, only 24.9% of the National Health Interview Survey respondents engaged with patient portal tools in 2017 [10]. Black Americans were less likely to activate their portal account and log-in and even less likely to use portal tools (12.2% of the Black National Health Interview Survey respondents) such as appointment scheduling, regardless of income or education [10,11]. Among Black Americans who are not registered for portal access, some barriers to portal use include a lack of support (ie, technological support and interaction with the treatment team); concerns for the loss of privacy, which are likely influenced by historical injustices within the health care system; and reduced personalization of care [12,13].

COVID-19 and the need to implement physical distancing to limit the spread of the associated virus have reduced the availability of in-person visits for primary and nonemergent care since early 2020 [12]. As of the second quarter of 2020, office-based visits were reduced by 50.2% [14]. In-person preventive care, chronic disease follow-up, and visits related to other primary care concerns can often be adapted to virtual care, although the frequency of routine assessments might change [14,15]. In place of in-person visits, primary care providers have increasingly used telemedicine and secure messaging to meet and communicate with their patients [12,14,15]. For example, one large primary care system asked 59% of its primary care staff to work from home by the end of March 2020, resulting in secure messaging to and from their 58,000 patients to increase by 41% and 51%, respectively [15]. In addition, telemedicine is also likely to increase the overall utility of patient portals because an increasing number of portals allow scheduling of telemedicine visits, access to the visit summary, and information on any recommended follow-up steps [5,6,15]. Following in the footsteps of federal agencies (ie, Medicare), private insurance companies have recently responded to the COVID-19 pandemic by lifting previous restrictions on telemedicine and by reducing or eliminating co-pays for telemedicine visits [12]. Although telemedicine is more accessible, the sudden shift to it has decreased the total number of appointments in primary care by 21.4%, according to the IQVIA (formerly Quintiles and IMS Health, Inc) National Disease and Therapeutic Index [14]. The frequency of assessments of cardiovascular risk factors and the prescription of new medications have also been reduced [14]. As Black Americans are already less likely to schedule office-based preventive care visits with their primary care provider [11,16], the need to digitize routine visits presents another barrier to scheduling, assessment, and treatment [14]. Digitization of health care will likely continue to grow even beyond the COVID-19 pandemic; thus, the importance of effectively connecting Black Americans to digital health care services is overwhelmingly necessary [17].

### Objectives

We used focus groups to understand potential barriers to optimized care for conditions disproportionately affecting Black Americans, and participants repeatedly noted the impact of using portals on their care. The perceived utility and value of patient portals were often integral to participant experiences with care and their relationships with providers, health care institutions, and their own health, as detailed herein.

## Methods

### Study Design

Self-identified Black American residents aged 30-60 years with regular access to primary care were recruited nationally using Craigslist and Research Match advertisements. Recruitment continued until thematic saturation [18] was reached. Interviews were conducted remotely through Webex (Cisco Systems) in April and May 2020, with both the study team and participants located in their own environments. The participants were sorted into focus groups on a first-come-first-serve basis. Individual phone calls were implemented to answer any participant questions, collect demographic data, and obtain verbal informed consent per the Oregon Health & Science University Institutional Review Board exemption guidelines.

Each interview was an hour long, with a maximum of 5 participants and 2-3 facilitators. One facilitator was responsible for primary facilitation and asking probing questions and at least one facilitator took notes. The focus groups began with the facilitators describing their role and any personal connection to the research and reminding participants that the information disclosed in the interview would remain confidential. The participants then introduced themselves and provided a first name or nickname for use in the focus group to engage with other participants; no other personal identifiers were provided during the session. The subsequent discussion was participant led. At least one facilitator self-identified as Black in every session. Interviews were recorded with video and audio, transcribed verbatim, and deidentified by the primary facilitator.

### Data Analysis

Patterns within the data were analyzed and identified through inductive thematic analysis using NVivo software (version 12.0; QSR International) [19]. An intercoder reliability analysis was performed using percent agreement among the raters [20]. Respondent validation was not used because of the quick evolution of the COVID-19 pandemic and the onset of nationwide protests that have involved many in the Black community and may therefore affect perspectives on health disparities or COVID-19 transmission and add additional participant burden [21]. The development of the interview guide was informed by grounded theory (Table 1). Study design and interview analysis were reported using the Consolidated Criteria for Reporting Qualitative Studies 32-item checklist (Table S1 in Multimedia Appendix 1) [22].

**Table 1:** Interview guide for 8 remote focus groups.

Prepared question	Interview numbers
Briefly describe a typical appointment with your primary care provider.	Interviews 1-3
Discuss the relationship between you and your primary care provider.	Interviews 1-8
When you have an appointment, how are recommendations or other health information conveyed to you?	Interviews 1-8
What are some health conditions that you think Black Americans are the most at risk of developing?	Interviews 1-3
Imagine that your primary care provider offered a new treatment or drug designed solely for Black Americans. How would you respond?	Interviews 1-3
If your primary care provider offered you a holistic plan to treat, for example, high blood pressure, including drugs, physical activity, and nutrition, would you be receptive to this? Why or why not?	Interviews 4-8
What are your concerns in the prevention and care of COVID-19?	Interview 4 and Interviews 6-8

### Theoretical Framework

A total of two theoretical frameworks were integrated to direct the discussion of emergent themes related to patient portal use among the participants: the technology acceptance model (TAM) and the health belief model (HBM) [23]. The TAM posits that the perceived usefulness and ease of use of a technology determines attitudes toward and actual use of that technology [23]. The HBM is used to predict health behavior and posits that individuals with high perceived personal risk of disease are more likely to seek health information and engage in health behaviors [24]. The integration of these models may predict the likelihood of adopting portal technology (TAM) to support an individual’s health awareness and provider communication and their engagement in healthy behaviors (HBM), including the adoption of preventive measures and treatment [25].

### Data and Materials Availability

The data that support these findings are available on request from the corresponding author, NPB; the data are not publicly available because of ethical restrictions.

## Results

### Overview

Eight focus groups engaged 29 participants (22 women) with a mean age of 40 (SD 8) years. Intercoder reliability was found to be in ≥95% agreement, such that secondary codes were not used in the analysis. Of the 29 participants, 22 (76%) discussed experiences related to patient portals. From the inductive thematic analysis, four themes emerged regarding patient portal use among Black Americans in primary care: optimization of care, patient empowerment, patient-provider communication, and patient burden. Table 2 provides the frequency of these four themes, and in the *Optimization of Care, Patient Empowerment, Patient-Provider Communication,* and *Patient Burden* sections, we provide some of the most salient individual examples that emerged from discussion in each of these themes.

**Table 2:** Frequency of identified themes in 8 focus groups.

Theme	Frequency values^a^
Optimization of care	29
Empowerment	15
Patient-provider communication	34
Patient burden	9

^a^Frequency was calculated as the total number of thematic endorsements of a particular code by an interviewee in any number of interviews.

### Optimization of Care

Most participants described patient portals as a tool to keep both the doctor and patient informed and organized, as well as to connect with the treatment team. For many, patient portals are integrated into the appointment routine and are considered a *go-to* tool for patients before and after each visit. Before the appointment, these portals were used for scheduling, requests for tests, insurance navigation, and communications. Scheduling is often the first point of contact for patients:

[The portal is] a really convenient tool especially for this day and age. I think that the doctor is more accessible that way. It’s a lot harder to have to schedule an appointment and have to go [in-person]. And then if something gets in the way and you have to reschedule, it could all be delayed.

Participant 4, group 1, Portland, Oregon

After scheduling, the portal helped patients to prepare for their next visit. As noted by 1 participant, physicians may respond differently to requests for tests. For this participant, use of the portal before appointments complemented her own engagement with her health, as well as her new physician’s willingness to work with her to understand her symptoms:

[The portal is] typically how I get my information...When I was desperately trying to find out what is wrong with me, I was always going online or finding something that I thought I might have. Interestingly enough, I would email my primary care physician via the web portal, and he would say “okay, well we’ll test you for this or test you for that...” My last experience before I switched primary care physicians was most harrowing: I didn’t feel heard, and I’m sure a lot of people have had this experience as well where they say “oh, you have this so, here, get this prescription,” or “do this,” or “do that.” So, it’s been quite a journey to finally find a diagnosis for what I have...I like [my new physician’s] approach because he is kind of like “let’s rule it out” versus, you know, “let me figure it out or let me tell you what I think you have....”

Participant 23, group 4, Fontana, California

Communication with the treatment team before the appointment reduced the possibilities of the participants forgetting or becoming too nervous to address specific health concerns:

I think you just have more time when you’re on the computer to think of what you really [want to] tell the doctor....

Participant 3, group 1, Denver, Colorado

During appointments, the participants reported the efficiency of having personal and family health histories centralized and readily available to the treatment team through their EHR:

[The portal] also makes things quicker because [the doctor] could just be like “alright from your last visit I see this, and let’s go ahead and move forward with that.” I think because the doctor already knows what they’re dealing with it probably makes it easier for them to make a more informed decision about [my] care... [When I] switch doctors it kind of feels like starting over...I think it helps having these uniform systems [and patient] information in the portal so [the new doctor] can at least catch up.

Participant 4, group 1, Portland, Oregon

After the appointment, treatment teams often contacted patients first (through phone, text, or secure email) for next steps. Participant access to portals for after-visit summaries and treatment recommendations (eg, prescription dosages and blood assay results) streamlined visit follow-up. Some platforms allowed participants to make payments and view insurance information:

[The portal is] my best good friend, medically speaking, because it’s what I prefer to use to check my results. [My provider] also follows up with conversations via the portal, so I use the portal quite a bit...from payment options, to referrals, to the information she conveys to me—any recommendations she makes [when] we are no longer face to face, that’s my primary go-to.

Participant 13, group 3, Pasadena, Maryland

Centralized patient health records, accessible to both the treatment team and the patient, limited redundancies and mistakes:

The portal is a very simple system, but I’m very thankful for that. [My primary care physician and I] can go back-and-forth and then if she tells me a certain dosage, I can just look on the portal—“oh what did she say?”

Participant 15, group 6, Staten Island, New York

For participants with relatively few health concerns, portal use reduced the number of appointments because test results and prescriptions are accessible on the web:

We use the portal a lot; sometimes I’m like three or four back-and-forth. In my mind this would’ve been a doctor’s visit back in the day, but we go back-and-forth.

Participant 15, group 6, Staten Island, New York

I rarely have any other visits in-between. I like being able to go get my physical and then almost instantaneously get those results...delivered to me via the portal.

Participant 17, group 5, Nashville, Tennessee

The participants generally appreciated the integration of portals into their treatment:

I remember the time that you had to ask them for your results and stuff like that. I think it adds [to my treatment] because then you already have a sense of—sometimes before the visit—of things you’re going to talk about. So, I think it’s more helpful and empowering for patients to have that access.

Participant 14, group 4, Portland, Oregon

### Patient Empowerment

Distrust was a major topic in the discussion of treatments for diseases that disproportionately affect Black Americans. Distrust was founded primarily on historical, personal, and media-based accounts of malpractice. Providers ignoring medication allergies or preferences was a commonly reported cause for distrust and switching providers. Under these circumstances, portals may facilitate the transfer of records. Patient portals also play a unique role in mitigating distrust, as participants described the importance of personal and family health histories—instead of, or in addition to, racial identity—in the consideration of a new treatment or therapy:

I’ve been with my primary care [provider] for years and years and years and I’ve been very vocal about what my concerns are, my family history, and my culture, and I’m already putting that stuff out there. [If] they come back to me like “oh, I agree with you, and per this research...,” I would be more receptive, because it’s like, “okay, you actually heard where I’m coming from. And you’re listening to what my concerns are and why I have these concerns, and you’re trying to work with me to create a solution.” But if it’s definitely from a place of privilege where it’s like “well, you know, you Blacks, the fried chicken and you got—” it’s like “oop, sorry, new doctor! That’s what you’re not going to do.”

Participant 23, group 4, La Mesa, California

This experience highlights the desire for personalized interactions among the participants and the importance of providing educational resources that connect previous discussions between the patient and provider to any new treatment recommendations. Educational resources were often provided digitally after appointments through secure messaging or portal-based visit summaries. Others obtained this information on their own through internet searches.

User-friendly portals also empowered the participants to discuss sensitive matters more transparently and to engage in their own health care more effectively. Most portal users reported benefiting from additional control of their appointments, although some participants viewed the portals as a hurdle between themselves and their provider rather than an additional avenue for communication (see *Patient Burden* section). Thorough reporting of health concerns through the portal was especially useful before the visit because crossing the digital divide served as an icebreaker in what can sometimes be timid interactions with the treatment team:

[The portal] lends a platform for being more honest and I think people in general are less uninhibited online. So, I think if you’re a patient you’re probably going to be a bit more forthcoming about what your symptoms are, or what you’re experiencing, or what’s going on in order for the doctor to better be able to assess what’s happening...I think we’ve all been in those situations at the doctor’s office and it’s awkward especially if it is a new doctor and you don’t know them really well. So, for me, I feel the need to be more detailed as well, because it is through text so I’m trying to make sure they can follow.

Participant 4, group 1, Portland, Oregon

The participants also stressed the importance of documenting health concerns on the portals to reduce in-person anxieties that may arise when under time pressure:

I agree with what [Participant 4] said...I just have more time to think it out, and can write it down, and really get maybe even better feedback. Or, at least feedback you can look back on and tell somebody else if you want a second opinion.

Participant 3, group 1, Denver, Colorado

I didn’t use [the portal] as much before because I thought it was kind of impersonal and dismissive... [Now I don’t] have to store [health concerns] in my mind and wait for an answer, because maybe I was a little bit anxious about whatever was going on with me...it’s helped take some of the anxiety out of me, especially as I started to develop a relationship with my new primary care physician and the nurse.

Participant 23, group 4, Fontana, California

Other anxieties potentially resulting in the participants forgetting or dismissing relevant points during appointments were commonly attributed to distrust based on experiences of being pressured by members of the treatment team or intimidation by a doctor’s demeanor.

According to the participants, physician accountability reciprocated patient accountability with regard to portal use. Many participants reported experiences in which their primary care providers would give the impression, either subtly or explicitly, that they did not have time for concerns beyond those routinely addressed in annual visits. The participants often avoided provider dismissiveness when they expressed their concerns through the portal before the visit:

I like to have [my health concerns] in writing and I feel like it’s going to hold the doctor more accountable having it in writing...the doctor gets back to you quicker [over the portals].

Participant 3, group 1, Denver, Colorado

One participant highlighted this theme in her experience of advocating for the health of her mother, an older adult. In this example, her mother’s treatment team was described as neglecting the patient, but the information stored in the portal served as a reference for her family members. Using her proxy access to her mother’s up-to-date portal, the daughter was able to contact the treatment team and make the necessary requests for tests that had previously been neglected:

When [my mom] was dealing with the MediCal, the people were overworked and underpaid...so I would go into my mother’s portal and health insurance and I would look for the doctors that she needs and then I would basically spell out everything for them... I’d write down dates and everything that I did so that they can’t turn around and say “oh, I forgot all this...” I definitely had to be the proactive one to go after them to show them if they were being not only insensitive but also they were giving information which was basically the opposite of what they said before. They were saying she has kidney issues and her hemoglobin [A1]C is elevated but then they didn’t follow up with her for four months. And I would say, “how didn’t you follow up when her diabetes is out of control?” But then you’re saying “you have to play a role; you have to take this medication...” I was like “that’s just not right. [There] was a huge void and a huge gap, and it worried me...It helps me understand more why anybody, but especially Black people are with the highest rates of diabetes, kidney disease, and amputations, and problems and not getting care...I can be here for [my mom], but a lot of people don’t have that because they’re working, or their kids don’t live with them, or they [just] don’t know.”

Participant 18, group 6, San Diego, California

As portals provide a way to conquer the digital divide, physicians may also be encouraged to obtain second opinions and other professional insights more readily. Thus, patient portals may increase transparency for both patients and their providers:

I imagine that [nervousness] is similar for the doctor, where if they’re in a face to face visit with you, it might be more awkward if they need to Google something or call a colleague. But if they’re not face to face with you, it does give them a little more time and anonymity and in being able to go consult with somebody if they need to.

Participant 4, group 1, Portland, Oregon

Health history stored on the portal underscored the value of transparency for increasing care satisfaction and patient engagement. Physician access to health records through EHRs tailored primary care visits to the specific health history of the patient. The ease of access to health information, such as test results and family health histories, complemented participant efforts to maintain accountability over their personal health outcomes:

I have, you know, parents, family health issues, and I want to make sure I’m not impacted. So, I try to stay a little more on top of things...I am a little more diligent.

Participant 15, group 6, Staten Island, New York

Reported lifestyle changes were most often dietary or exercise-related. Commonly reported steps to counteract increased health risk were typically related to conditions that participants knew disproportionately affected Black Americans. Participants were specifically aware of racial disparities in the prevalence of diseases such as COVID-19, cardiovascular disease, and diabetes, and accountability for health was usually attributed to the participants’ personal and family history of disease. Chronic disease was common among participants and their family members, and EHRs documented and facilitated discussions of these health concerns. The accessibility of health records made possible through most portals enabled a level of patient accountability necessary to address increased health risks:

I don’t have to feel like I have to play politics, particularly with my PCP... [Regarding portal use]. Usually I initiate, but they’re willing to give me the information that I need or want and allow me, without trouble, to make decisions. They don’t treat me like oh you’re the patient and you can’t make the decisions...

Participant 18, group 6, San Diego, California

Some participants wanting a new provider felt too invested to find one if their family health records were stored locally because sharing this information with a new physician might be time consuming, as previously noted. Digital records enabled participants to change physicians without communicating family and personal health histories anew (if transferring to an interoperable site) and to obtain second opinions easily.

### Patient-Provider Communication

Some participants expected patient portals to reduce personalization of care but instead found that the portals supplemented their interactions with their treatment team. The accessibility of portal communications enabled many participants to address their concerns more thoroughly and accurately, increasing their engagement with their treatment plans. Participants with negative experiences regarding the portal were often dissatisfied with their treatment team at the time and were sometimes seeking a new primary care provider. Overall, communication through portals mirrored communication patterns already established between the patient and their provider (eg, efficient and accommodating or terse and impersonal):

[The portal is] a double-edged sword, right?...The best way of thinking of it I think is that [if] I’m texting with my doctor, you can either give me a short answer or just say “yes/no” without any other follow up or results. And there’s other instances that people use that as a “hey, I saw your message, I actually wanted to give you a call. After I get off the phone with you, I’m going to send you another message and we’ll check in...” So, it all depends really more so on the doctor. If they use that as another touch point to stay in touch, that’s good.

Participant 23, group 4, La Mesa, California

Assessments that were performed that day [are] delivered to me via [the] portal...I’ve also given them permission to notify me by email and by text....I’ll get a [text] message, that might say “you have a new test result” and I like that, and I find it very helpful...I like the connectivity...Whenever I’ve had to reach out to my primary care physician, I get a pretty good return rate. You know, usually within 24-48 hours if I ever have any questions....

Participant 17, group 5, Nashville, Tennessee

Provider response time was generally important to participants, as was the thoroughness of response:

Pretty rapid response time but very minimal in the communication. Just very, like, terse, which is what it is. That was the only method of communication.

Participant 16, group 6, Portland, Oregon

Although communication through patient portals was common, portal use failed to supplement in-person communication for some participants. Ineffective communication patterns and delegation of follow-ups from the provider to other treatment team members (eg, medical assistant or nurse) compromised satisfaction with portal-based communications. Portal use hindered effective communication in at least two scenarios. First, when a phone call was still necessary to fill the request or answer a question:

It’s a longer process for them to get into the portal when I can just call and say what I have to say, instead of waiting a week to get an answer.

Participant 22, group 5, St. Louis, Missouri

Second, when digital responses from the provider are delayed, insufficiently detailed, or confusing:

When the replies are delayed, that kills me the most. Like, “hey, I sent a message on Monday. Here it is, now Monday the following week. What’s up?”

Participant 23, group 4, La Mesa, California

For many participants, the nurse or medical assistant was in charge of much of the digital communication with the patients. Delegation to other treatment team members limited digital communication with primary care providers for some participants compared with those who received secure emails and phone call check-ins from their provider. Although some participants were indifferent to delegated communications, personalized messages from the primary care provider were especially appreciated. Weekend, after-hours, and between-appointment communications represented a level of care that exceeded expectations.

### Patient Burden

Portals provide a variety of tools but also rely on the patient to take advantage of these tools outside of the appointment. Some participants had trouble adapting. In addition to technological setbacks, some participants described the portals as an extra step to contact their treatment team, especially in cases where a phone call is inevitable. The sophistication of the portal interface may also play a role in burdening the patient because some participants reported that messages are not forwarded to their email, requiring them to log in proactively to check their inbox. A Portland, Oregon, participant recalled that she had experienced this issue with her past primary care provider:

Everything was communicated through a portal. But I had to actively go into the portal to see if there were communications. It wasn’t like if she sent me a message and that would show up in my personal email and then I would know—I had to really be looking and waiting.

Participant 16, group 6, Portland, Oregon

Many participants were aware of the lack of interoperability among portals and were frustrated at having to recount family health history to new providers when switching insurance networks or moving:

I bring everything back to my primary care doctor. There’s not a lot of communication between specialists. They do have their own little charts [records] and that can be hard.

Participant 18, group 6, San Diego, California

In addition to changing appointment routines to adapt to the portal, some participants with long-term relationships with their providers noted the burden that portals placed on hospitals and treatment team members to learn how to implement them in their practice. This physician burden resulted in at least 2 participants losing their long-term primary care provider when the portal was introduced at their place of care:

This doctor retired and then, I don't know, he said he was kind of almost forced out a few years back when everything was going digital and he didn’t. He’s on the older side so he didn’t kinda learn the new system and stuff...I did have to switch to someone in the meantime, and I got a referral for her from a family member. But, yeah, she wasn’t as accommodating I’d say, and that’s why I went back to my first doctor.

Participant 5, group 2, Portland, Oregon

The participant chose the existing rapport she had with her very accommodating provider over a digitalized form of their relationship. Another participant noticed that she rarely received responses to secure messages and wondered if her treatment team had any training on the use of portal-based communications.

## Discussion

### Principal Findings

In the past, structural barriers have limited the initial portal sign-on by Black Americans [11-13]. In our focus groups with Black primary care patients who do use patient portals, the most frequently referenced themes were (1) optimization of care, (2) patient empowerment, (3) patient-provider communication, and (4) patient burden. The two most common themes related to patient portal use in our focus groups, namely optimization of care and patient empowerment, suggest that experiences with patient portals are largely positive. The influence of patient portal use on patient-provider communication and patient burden was generally contingent on previously established communication patterns, as well as support from the treatment team. Similarly, previously identified barriers to portal use among racial minorities who had little or no history of portal use include expectations regarding technological support and the quality of portal-based communications [13].

### Technology Acceptance and HBMs

Applying the TAM, the emergent themes in our focus groups suggest that when the perceived usefulness and ease of use of patient portals improve, attitudes toward portals improve and so does the actual use of patient portals among Black Americans [23]. The perceived usefulness of patient portals for optimized care among our participants is further explained by the integration of the HBM with the TAM. According to the integrated model, individuals with high perceived susceptibility to disease and high health consciousness are more likely to adopt technology that fulfills health information and communication needs or that supports health-seeking behavior [24,25]. Meeting such needs in a way that is perceived as both easy and beneficial subsequently influences and reinforces positive attitudes toward and increased use of health technologies [23,25]. Susceptibility to disease was a common concern among the interview participants. In the face of historical mistreatment and ongoing bias against Black Americans in health care settings, portals may offer Black patients control over their appointments while providing tools for personalized care to mitigate bias. This finding is consistent with a March 2020 survey [26] showing that Black Americans who viewed COVID-19 as a minor personal health threat (as opposed to no threat at all to personal health or a major threat for which in-person assessments may be necessary) had the highest use of telemedicine as a result of the pandemic compared with White respondents, despite anticipated barriers. This may be due to increased perceived susceptibility to COVID-19 among Black Americans, resulting in an abundance of caution regarding in-person visits compared with White Americans with the same level of perceived threat [26].

Although telehealth may replace some elements of in-person visits, the results from this study suggest that portals may be relied upon for the optimization of care before, during, and after appointments when used by Black Americans. Once a telehealth or in-person appointment is made, the personalization of care may still be limited by implicit bias against Black Americans. Patient portals may help reduce this disparity by holding providers accountable for addressing specific health concerns documented on the web, rather than requiring patients to quickly recall and disclose sensitive information in person. Patients run the risk of making mistakes, feeling uncomfortable, and even being traumatized by having to repeat health information every time they meet with a provider when their personal health information is decentralized (spread across multiple local EHRs) or if the patient lacks an EHR altogether [27]. Black patients may feel this distress more poignantly [27], compounding existing distrust that is common among Black Americans in health care settings because of interpersonal and institutional racial discrimination [17]. Furthermore, centralized and up-to-date EHRs facilitate provider treatment recommendations based on personal and family health records and on environmental factors that have been documented, thereby improving health outcomes [1].

### Looking Beyond Race

In contrast, race is often leveraged to assess risk in the treatment of Black Americans for conditions that they are at heightened risk for developing severe symptoms, including COVID-19 [28,29]. Race-based medicine and common risk assessments that use algorithms adjusted by a patient’s race are present in cardiology, endocrinology, nephrology, urology, oncology, obstetrics, and other specialties [29]. This practice of operationalizing racial and ethnic categories results in the differential prescription of specialty services (including assessments, treatments, and major surgical procedures) for Black patients and other racial or ethnic minorities [29]. The prescriptive use of race in clinics may exacerbate inequalities and perpetuate implicit bias at health care institutions [17,29]. This contrasts with the use of race in descriptive statistics, which are vital for identifying disparities in health and for beginning to understand the etiology of disease (including systemic racism) and subsequent downstream effects (such as reduced access to care or increased psychosocial and environmental stressors) [8,29].

The impacts of race-based risk assessments for Black Americans at clinics include under- or overprescription of pain medications, reduced options for life-saving surgeries such as cesarean sections during childbirth because of preoperative risk assessments, less aggressive screenings for bone disorders and some forms of cancer because of lower risk of developing the condition (eg, breast cancer), and increased or decreased likelihood of intervention resulting from a decreased likelihood of survival (eg, rectal cancer treatment, in which doctors may not recommend treatment to patients who are unlikely to survive and recover) [29]. Although risk-assessment algorithms intend to increase efficiency in diagnosis and decrease costs, without proper scrutiny and understanding of the distinctions between biological mechanisms of disease and social determinants [8,17], adjustments by race might be arbitrary or even harmful or fatal [29]. In other words, racial identity is certainly not a replacement for a patient’s health records. In our study, the focus group participants generally felt empowered by access to their own health information, including test results. Provider familiarity (or lack thereof) with family health histories was important to the participants, especially those considering looking for a new provider. This priority is consistent with the outcome of a community-based intervention for cancer-risk perception in Black Americans that emphasizes family health history to assess objective risk [30]. After the intervention, wherein family health histories were disseminated to the participants, objective and subjective risk levels matched in most of the participants [30]. Contextualizing subsequent treatments for the patient would likely require further collaboration with the patient to understand the barriers to effective care.

Portal-based communications emphasize the communication patterns already established between a patient and their provider. To this extent, portals provide the treatment team with additional opportunities for exceptional and tailored care. Care centered on the unique needs and experiences of the patient may be an expectation for many patients, particularly those with access to their own health records [1], but Black patients experience unfair bias in medical settings [17,29]. Predisposition to chronic disease and implicit bias in the treatment of such diseases place pressure on Black Americans seeking care. Indeed, recent work suggests that Black patients in primary care may feel that they must take great care to protect themselves from the health effects of structural racism while also confronting family history of disease through lifestyle choices and engaging more with their health [26,28]. By matching patient accountability with a level of care that exceeds expectations, regular portal use by patients and their providers can reduce bias and therefore alleviate some of the burden of disparity from the shoulders of Black patients [9]. Specifically, setting realistic and informed goals and then checking in proactively with the patient between appointments may address barriers in primary care with tools and information readily accessible on patient portals [6,9,31].

### Strengths and Limitations

The potential study limitations include generalizability to populations lacking health coverage, access to internet-compatible devices, or regular access to patient portals. The themes discussed in this paper should be confirmed using quantitative methods, especially considering the increased use of telemedicine over the last year [12,14,15]. Web-based recruitment, as well as the web-based format of the interviews, also limits generalizability to individuals who are less computer savvy. The participants were aged between 30 and 60 years because of the requirements of the parent study of this investigation. Age is a limitation because older patients are likely to interact with their treatment team more often and may have more visits to manage. Despite the benefits of portals in managing care among providers, older populations may use portals less often than our participants and would require even more technological support and coordination between providers and other members of the treatment team [13,14]. As lack of technological support contributes to lower use of patient portals among Black Americans generally, our focus groups show how increasing support, communication, and adoption of patient portals may provide opportunities for better health care among Black patients.

## Appendix

Multimedia Appendix 1 Consolidated Criteria for Reporting Qualitative Studies 32-item checklist.

## Abbreviations

EHR: electronic health record
HBM: health belief model
TAM: technology acceptance model
